# Impact of early mean arterial pressure level on severe acute kidney injury occurrence after out-of-hospital cardiac arrest

**DOI:** 10.1186/s13613-022-01045-1

**Published:** 2022-07-18

**Authors:** Vincent Dupont, Anne-Sophie Bonnet-Lebrun, Alice Boileve, Julien Charpentier, Jean-Paul Mira, Guillaume Geri, Alain Cariou, Mathieu Jozwiak

**Affiliations:** 1 Centre Hospitalier Universitaire de Reims, University Hospital of Reims, Reims, France; 2 French Clinical Research Infrastructure Network, Investigation Network Initiative - Cardiovascular and Renal Clinical Trialists (F-CRIN INI-CRCT), Reims, France; 3 British Antarctic Survey, High Cross, Madingley Road, Cambridge, CB4 0ET UK; 4 Département de Médecine Oncologique, Gustave Roussy, 94805 Villejuif, France; 5 Assistance Publique – Hôpitaux de Paris, Hôpitaux Universitaires Paris-Centre, Hôpital Cochin, Service de Médecine Intensive Réanimation, 27, Rue du Faubourg Saint Jacques, Paris, France; 6 Université de Paris, Paris, France; 7 Service de Médecine Intensive et Réanimation, Assistance Publique-Hôpitaux de Paris, Hôpital Universitaire Ambroise Paré, Boulogne-Billancourt, France; 8 Université Paris-Saclay, Gif-sur-Yvette, France; 9 INSERM, UMR1018, Centre de Recherche en Epidémiologie et Santé des Populations, Villejuif, France; 10 INSERM U970, Paris-Cardiovascular-Research-Center, Paris, France; 11 Paris Sudden-Death-Expertise-Centre, Paris, France; 12 AfterROSC Network Group, Paris, France; 13 Centre Hospitalier Universitaire l’Archet 1, Service de Médecine Intensive Réanimation, Nice, France; 14 Equipe 2 CARRES, UR2CA Unité de Recherche Clinique Université Côte d’Azur, Université Côte d’Azur, Nice, France

**Keywords:** Out-of-hospital cardiac arrest, Post-resuscitation shock, Ischemia–reperfusion syndrome, Acute kidney injury

## Abstract

**Background:**

The optimal early mean arterial pressure (MAP) level in terms of renal function remains to be established in patients with out-of-hospital cardiac arrest (OHCA). We aimed to evaluate the association between early MAP level and severe acute kidney injury (AKI) occurrence in patients with OHCA.

**Results:**

In 568 consecutive patients, the percentage time spent below a predefined MAP threshold and the corresponding area below threshold (ABT) were calculated from continuous MAP measurement. Both MAP-derived variables were calculated for different MAP thresholds (65, 75 and 85 mmHg) and time periods (the first 6 and 12 after ICU admission). 274 (48%) patients developed severe AKI defined as stage 3 of KDIGO. Both ABT and percentage time were independently associated with severe AKI, regardless of the MAP threshold and time period considered. Highest adjusted odds ratios for developing severe AKI were observed while considering the first 6 h period. Within the first 6 h, every 100 mmHg-h increase in ABT under MAP thresholds of 65, 75 and 85 mmHg increased severe AKI risk by 69% (OR = 1.69; 95% CI 1.26–2.26; *p* < 0.01), 13% (OR = 1.13; 95% CI 1.07–1.20; *p* < 0.01) and 4% (OR = 1.04; 95% CI 1.02–1.06; *p* < 0.01), respectively. Every 10% increase in percentage time spent under MAP thresholds of 65, 75 and 85 mmHg increased severe AKI risk by 19% (OR = 1.19; 95% CI 1.06–1.33; *p* < 0.01), 12% (OR = 1.12; 95% CI 1.04–1.19; *p* < 0.01) and 8% (OR = 1.08; 95% CI 1.02–1.14; *p* < 0.01), respectively.

**Conclusions:**

Both severity and duration of early arterial hypotension after ICU admission remained associated with severe AKI occurrence while considering a MAP threshold as high as 85 mmHg after OHCA.

**Supplementary Information:**

The online version contains supplementary material available at 10.1186/s13613-022-01045-1.

## Background

Out-of-hospital cardiac arrest (OHCA) is a major public health challenge with a global incidence of 55 per 100,000 person-years and a survival rate of 8.8% after hospital discharge [1]. Arterial hypotension after OHCA is multifactorial and is a consequence of post-resuscitation shock (due to the ischemia reperfusion syndrome), cardiogenic shock and/or pre-existing cardiac pathology. Whatever the cause, arterial hypotension is associated with multiple organ failure and increased morbidity and mortality after OHCA [2–4]. Thus, interventions to prevent and/or manage organ damage are direly needed to improve the poor outcome in this population.

Acute kidney injury (AKI) occurs in 10–80% of patients with OHCA and requires renal replacement therapy in one-third of patients [5, 6]. Severe AKI is associated with long-term occurrence of chronic kidney disease as well as poor neurological outcomes and increased mortality [6–9]. It has been previously shown that risk factors of severe AKI in patients with OHCA were age, gender, resuscitation duration, public setting, initial rhythm and post-resuscitation syndrome [6]. However, none can be modified by specific therapeutics once the patient is admitted in intensive care unit (ICU), which considerably limits the development of interventional strategies for the clinicians [6].

The optimal mean arterial pressure (MAP) target remains to be established in patients with OHCA [10]. However, higher MAP level was found to be associated with lower odds of renal replacement therapy in this population [11]. Here, we investigated the association between different MAP thresholds within the first 6 and 12 h of management after OHCA and the occurrence of severe AKI.

## Methods

### Patients

We performed a post hoc analysis of a previous single-center observational study of our group [6]. This study complied with the Strengthening of Reporting in Observational studies in Epidemiology (STROBE) checklist and was approved by the Ethics Committee of the Société de Réanimation de Langue Française (IRB number CE-SRLF12-384).

All consecutive comatose patients with OHCA admitted between January 2007 and December 2012 were included. We excluded (1) patients who were not treated with targeted temperature management (TTM) to enhance the generalization of our results [10]; (2) patients with history of chronic kidney disease because of their inherent higher risk of developing severe AKI; (3) patients who died within the first 48 h to avoid the competitive risk between death and severe AKI occurrence; and (4) patients for whom no MAP level was available, preventing full case analysis.

### Data collection

The following variables were prospectively collected: demographic characteristics, comorbidities, clinical and biological data, cardiac arrest location, time from collapse to basic life support and to sustainable return of spontaneous circulation (ROSC), initial rhythm, hypothermia management, therapeutics and ICU mortality.

We defined severe AKI as the occurrence of stage 3 AKI within the first 48 h after ICU admission according to the Kidney Disease Improving Global Outcomes (KDIGO) classification using both creatinine, urine output and renal replacement therapy (RRT) criteria. We focused on AKI occurrence within the first 48 h as we aimed to assess its association with early MAP level after ICU admission (as AKI developing later during ICU stay may rather be related to multiple factors, other than early arterial hypotension). Since premorbid creatinine is often not available, we considered the first creatinine level (measured at ICU admission) as baseline for all patients [6]. To ensure that admission creatinine did not constitute a large deviation from reality, we compared it to the estimated creatinine level assuming a MDRD = 75 mL/min/1.73 m^2^ [12].

### MAP-derived variables

MAP measurements started at the time of ICU admission and were obtained initially non-invasively and then invasively with an arterial catheter at the femoral or radial artery level. We considered the initial non-invasive MAP measurements in our analysis to account for the effect of MAP at the earliest possible phase of hemodynamic management. The percentage of non-invasive MAP measurements was calculated.

Both MAP levels and norepinephrine dosages were prospectively and continuously monitored using a dedicated software with a refreshing rate of two per minute (GE Healthcare Centricity, Chicago, IL, USA) during the whole ICU stay. Then, variables were extracted for offline analysis with a dedicated computer program using the R package DescTools (R package version 0.99.34, R Foundation for Statistical Computing, Vienna, Austria).

### Mean MAP value

For each patient, the program calculated the mean value of MAP for two time periods of interest (the first 6 and 12 h after ICU admission).

### Area below the MAP threshold

**Fig. 1:**
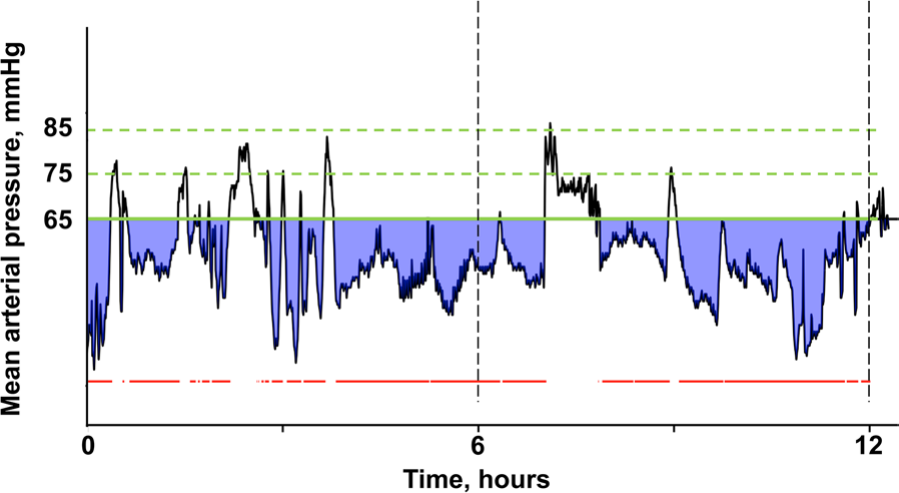
Definition of MAP-derived variables. For a given mean arterial pressure (MAP) threshold (green line), the time spent under the MAP threshold (represented by the red line) as well as the area below MAP threshold (ABT, purple area), which reflects both the time spent (duration of hypotension) under the MAP threshold and the magnitude of MAP derivation (severity of hypotension), were calculated. Percentage time and ABT were calculated for different MAP thresholds (65, 75 and 85 mmHg) and for different time periods (the first 6 and 12 h after ICU admission)

Because the analysis of the mean MAP alone does not allow to test the impact of the time and severity of arterial hypotension under different MAP thresholds on severe AKI occurrence, we designed a new data analysis method based on our granular dataset. For a given prespecified MAP threshold, the program identified for each patient the sections when MAP was continuously below the threshold considered and then calculated the area below threshold (ABT), i.e., the area between the MAP curve and the threshold line in these sections, which reflects both the time spent under predefined threshold (duration of arterial hypotension) and the magnitude of MAP derivation (severity of arterial hypotension) (Fig. 1). We calculated the ABT corresponding to three different MAP thresholds (65, 75 and 85 mmHg) for the two time periods of interest (the first 6 and 12 h after ICU admission) (Fig. 1).

### Percentage time under the MAP threshold

As we aimed to identify the most relevant MAP threshold in terms of severe AKI occurrence, and since the association between severe AKI occurrence and the ABT corresponding to one predefined threshold might be driven by the severity of arterial hypotension, we used the same methodology to calculate the percentage time spent under each MAP threshold. In this way, we could assess the association between severe AKI occurrence and the time spent under the three different MAP thresholds (65, 75 and 85 mmHg) for the two time periods of interest (the first 6 and 12 h after ICU admission), independently from the severity of arterial hypotension.

### Early management of patients

As previously described, our local practices included a strategy of early imaging diagnosis performed within the first 24 h after an immediate feasibility assessment by emergency medical services and ICU physicians [13]. Coronary angiogram was performed in all patients without obvious extracardiac cause of cardiac arrest, regardless of initial rhythm and electrocardiogram changes. A percutaneous coronary intervention was attempted if a culprit coronary lesion was considered as the cause of OHCA.

In addition to severe AKI, RRT could also be initiated for severe metabolic acidosis (pH < 7.20 and bicarbonate level < 20 mmol/L) and/or life-threatening hyperkalemia (K^+^  > 6 mmol/L with electrocardiographic findings suggestive of hyperkalemia) [6]. According to our local practices during the study period, TTM (33 °C) was performed in all patients using the forced cold air method within the first 24 h following admission.

### Statistical analysis

Normal distribution of continuous data was tested using the Kolmogorov–Smirnov test. Continuous variables were expressed as mean ± standard deviation or median [interquartile] and categorical variables were expressed as counts (percentages). Comparisons between patients with and without severe AKI were performed using Student *t*-test or Mann–Whitney *U*-test for continuous variables and Chi^2^ or Fisher exact tests for categorical variables. The impact of iodinated contrast injection on severe AKI occurrence was evaluated as a qualitative and also as a quantitative variable (number of investigations requiring iodinated contrast injection; i.e., coronary angiography or CT scan) [6].

To assess the association between mean MAP value and severe AKI occurrence, we performed different stepwise backward logistic regression models with one model for each time period of interest (the first 6 and 12 h after ICU admission). Receiver operating characteristic (ROC) curves (with 95% confidence interval, [CI]) were also generated to assess the ability of the mean MAP value within the first 6 and 12 h after ICU admission to predict severe AKI occurrence. The best threshold value was determined so as to maximize the Youden Index (specificity + sensitivity − 1).

To assess the association between other MAP-derived variables and severe AKI occurrence, we performed different stepwise backward logistic regression models with one model for each combination of MAP thresholds (65, 75 and 85 mmHg) and time periods (the first 6 and 12 h after ICU admission). Each model included either ABT or the percentage time spent under the MAP threshold.

For each multivariable model, we included the following covariates: age, gender, witness attendance, initial rhythm, epinephrine use during resuscitation, resuscitation durations, admission creatinine level, history of arterial hypertension and median norepinephrine dosage. The adjusted odds ratio (OR) and their 95% CI were calculated for all independent factors associated with severe AKI occurrence.

Statistical analysis was performed using XLSTAT version 2020.1.1 (Excel, Microsoft Corp, Seattle, WA). A *p*-value < 0.05 was considered statistically significant.

## Results

### Patients

**Fig. 2:**
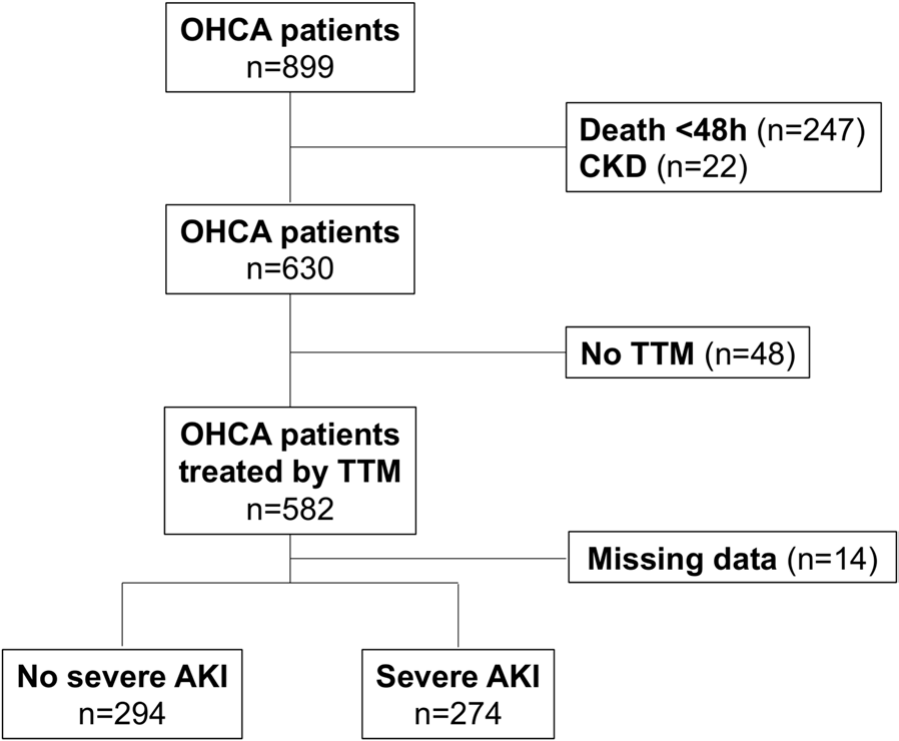
Flowchart of the study. *OHCA* out-of-hospital cardiac arrest, *AKI* acute kidney injury, *CKD* chronic kidney disease, *TTM* targeted temperature management

Among the 899 patients with OHCA admitted during the study period, 568 patients were included in the analysis (Fig. 2). The 14 patients excluded because of missing MAP values were not significantly different from the studied cohort (Additional file 1: Table S1). 404 (71%) were men and median age was 59 [49–71] years old. At ICU admission, mean MAP level was 68 ± 29 mmHg and blood lactate level was 5.0 ± 3.6 mmol/L. 208 (37%) patients received norepinephrine within the first 12 h, of those who required noradrenaline the mean dosage was 0.33 ± 0.49 μg/kg/min. Within the first 48 h, 274 (48%) patients developed severe AKI and 164 (60%) required RRT. Median ratio between admission creatinine and estimated creatinine levels was 1.05 [0.83–1.35], hence the use of admission creatinine neither modified the population with severe AKI within the first 48 h nor altered our results. All patients who required RRT within the first 48 h also met KDIGO stage 3 creatinine or urine output criteria, thus no patient was started on RRT for an indication other than renal failure without also meeting KDIGO 3 criteria. The number of investigations with iodinated contrast injection did not affect severe AKI occurrence (OR = 1.21, 95% CI 0.89–1.65; *p* = 0.22). Day-30 mortality rate was 58% and was significantly higher in patients with severe AKI than in those without (70 vs 46%, *p* < 0.01). The other patients’ baseline characteristics are summarized in Table 1.

**Table 1:** Patient characteristics according to the occurrence of severe acute kidney injury

Variables	All patients (*n* = 568)	Severe AKI		*p* value
		No (*n* = 294)	Yes (*n* = 274)	
Male, *n* (%)	404 (71)	219 (74)	185 (67)	0.04
Age, years	59 (49–71)	56 (47–69)	62 (50–72)	< 0.01
BMI, kg/m^2^	25 (23–28)	25 (22–27)	26 (23–29)	0.16
Hypertension, *n* (%)	219 (39)	108 (37)	111 (40)	0.89
Diabetes, *n* (%)	89 (16)	42 (14)	47 (17)	0.62
Witnessed CA, *n* (%)	491 (86)	262 (89)	229 (84)	0.05
Bystander CPR, *n* (%)	309 (54)	169 (57)	140 (51)	0.05
VF/VT, *n* (%)	324 (57)	188 (64)	136 (50)	< 0.01
Epinephrine use during CPR, mg	2.8 ± 3.6	2.2 ± 3.2	3.5 ± 3.8	< 0.01
Time from collapse to CPR, min	4.9 ± 5.5	4.2 ± 4.4	5.8 ± 6.4	< 0.01
Time from CPR to ROSC, min	17.8 ± 11.9	15.2 ± 10.0	20.6 ± 13.1	< 0.01
Iodinated contrast, *n* (%)	421 (74)	219 (74)	202 (74)	0.90
Coronary angiogram, *n* (%)	408 (72)	213 (72)	195 (71)	0.28
Admission creatinine level, μmol/L	109 ± 53	94 ± 37	125 ± 63	< 0.01
Admission lactate level, mmol/L	5.0 ± 3.6	3.9 ± 3.8	6.3 ± 4.0	< 0.01
Diuresis within the first 24 h, mL/kg/h	0.9 ± 0.8	1.7 ± 0.6	0.5 ± 0.6	< 0.01
Patients under norepinephrine, *n* (%)				
At 6 h	175 (31)	76 (26)	99 (36)	< 0.01
At 12 h	144 (25)	67 (23)	77 (28)	0.16
Norepinephrine dosage, μg/kg/min				
Within first 6 h	0.12 ± 0.36	0.07 ± 0.21	0.18 ± 0.47	< 0.01
Within first 12 h	0.11 ± 0.32	0.07 ± 0.23	0.15 ± 0.39	< 0.01
Day-30 mortality, *n* (%)	328 (58)	135 (46)	193 (70)	< 0.01

Data are expressed as mean ± standard deviation, median [interquartile] or number (%)

*AKI* acute kidney injury, *BMI* body mass index, *CA* cardiac arrest, *CPR* cardiopulmonary resuscitation, *VF* ventricular fibrillation, *VT* ventricular tachycardia, *ROSC* return of spontaneous circulation

### Mean MAP value and severe AKI occurrence

Within the first 12 h after ICU admission, MAP was non-invasively recorded during 19.4 ± 33.2% of time. Within the first 6 h and 12 h after ICU admission, mean MAP value was 91 ± 14 mmHg and 88 ± 12 mmHg, respectively, and mean MAP value was significantly lower in patients developing severe AKI within the first 48 h, regardless of the time period considered: 88 ± 14 vs. 94 ± 14 mmHg (*p* < 0.01) within the first 6 h and 85 ± 12 vs. 90 ± 12 mmHg (*p* < 0.01) within the first 12 h after ICU admission.

After adjustment for covariates, mean MAP value was not associated with severe AKI occurrence, regardless of the time period considered (Table 2, Additional file 1: Tables S2, S3).

**Table 2:** Association between mean arterial pressure level and severe acute kidney injury occurrence: summary of the different regression models

Variables	All patients (*n* = 568)	Severe AKI		Univariate analysis		Multivariate analysis	
		No (*n* = 294)	Yes (*n* = 274)	Unadjusted OR [95% CI]	*p* value	Adjusted OR [95% CI]	*p* value
First 6 h							
Mean MAP value, mmHg	91 ± 14	94 ± 14	88 ± 14	0.99 [0.95–0.98]	< 0.01	1.00 [0.98–1.02]	0.93
ABT MAP < 65 mmHg, 10^2^ mmHg-h	0.7 ± 0.2	0.3 ± 1.1	1.2 ± 3.0	1.34[1.16–1.54]	< 0.01	1.69 [1.26–2.26]	< 0.01
ABT MAP < 75 mmHg, 10^2^ mmHg-h	3.2 ± 6.5	1.8 ± 4.4	4.6 ± 7.9	1.09 [1.05–1.13]	< 0.01	1.13 [1.07–1.20]	< 0.01
ABT MAP < 85 mmHg, 10^2^ mmHg-h	9.2 ± 14.5	6.2 ± 11.6	12.3 ± 16.5	1.03 [1.02–1.05]	< 0.01	1.04 [1.02–1.06]	< 0.01
Percentage time MAP < 65 mmHg, 10%	0.9 ± 1.8	0.6 ± 1.4	1.3 ± 2.0	1.26 [1.15–1.38]	< 0.01	1.19 [1.06–1.33]	< 0.01
Percentage time MAP < 75 mmHg, 10%	2.4 ± 3.0	1.7 ± 2.7	3.1 ± 3.1	1.23 [1.10–1.16]	< 0.01	1.12 [1.04–1.19]	< 0.01
Percentage time MAP < 85 mmHg, 10%	4.2 ± 3.5	3.5 ± 3.5	4.9 ± 3.4	1.11 [1.06–1.16]	< 0.01	1.08 [1.02–1.14]	< 0.01
First 12 h							
Mean MAP value, mmHg	88 ± 12	90 ± 12	85 ± 12	0.96 [0.94–0.98]	< 0.01	1.01 [0.99–1.03]	0.50
ABT MAP < 65 mmHg, 10^2^ mmHg-h	1.6 ± 10.6	0.4 ± 1.6	2.8 ± 15.1	1.28 [1.14–1.43]	< 0.01	1.48 [1.21–1.81]	< 0.01
ABT MAP < 75 mmHg, 10^2^ mmHg-h	6.5 ± 21.1	2.9 ± 7.5	10.3 ± 28.9	1.05 [1.03–1.08]	< 0.01	1.07 [1.04–1.10]	< 0.01
ABT MAP < 85 mmHg, 10^2^ mmHg-h	18.2 ± 34.6	10.9 ± 21.0	26.1 ± 43.6	1.02 [1.01–1.03]	< 0.01	1.02 [1.01–1.03]	< 0.01
Percentage time MAP < 65 mmHg, 10%	0.9 ± 1.8	0.5 ± 1.4	1.3 ± 2.0	1.32 [1.19–1.44]	< 0.01	1.23 [1.09–1.37]	< 0.01
Percentage time MAP < 75 mmHg, 10%	2.5 ± 2.9	1.8 ± 2.6	3.2 ± 3.0	1.18 [1.12–1.25]	< 0.01	1.14 [1.06–1.22]	< 0.01
Percentage time MAP < 85 mmHg, 10%	4.3 ± 3.5	3.7 ± 3.5	5.1 ± 3.4	1.12 [1.07–1.16]	< 0.01	1.08 [1.02–1.14]	< 0.01

Variables are expressed as mean ± standard deviation. All logistic regression models included one of the MAP-derived variables (percentage time spent under the MAP threshold or ABT in each model), age, gender, witness attendance, initial rhythm, epinephrine use during resuscitation, resuscitation durations admission creatinine level, history of arterial hypertension and median norepinephrine dosage

*AKI* acute kidney injury, *ABT* area below threshold, *CI* confidence interval, *MAP* mean arterial pressure

A mean MAP value ≤ 90 mmHg within the first 6 h predicted severe AKI occurrence with a sensitivity of 63% (95% CI 56–69%) and a specificity of 61% (95% CI 54–67%). A mean MAP value ≤ 87 mmHg within the first 12 h predicted severe AKI occurrence with a sensitivity of 64% (95% CI 57–70%) and a specificity of 56% (95% CI 49–62%) (Additional file 1: Fig. S1).

### Area below the MAP threshold (ABT) and severe AKI occurrence

**Fig. 3:**
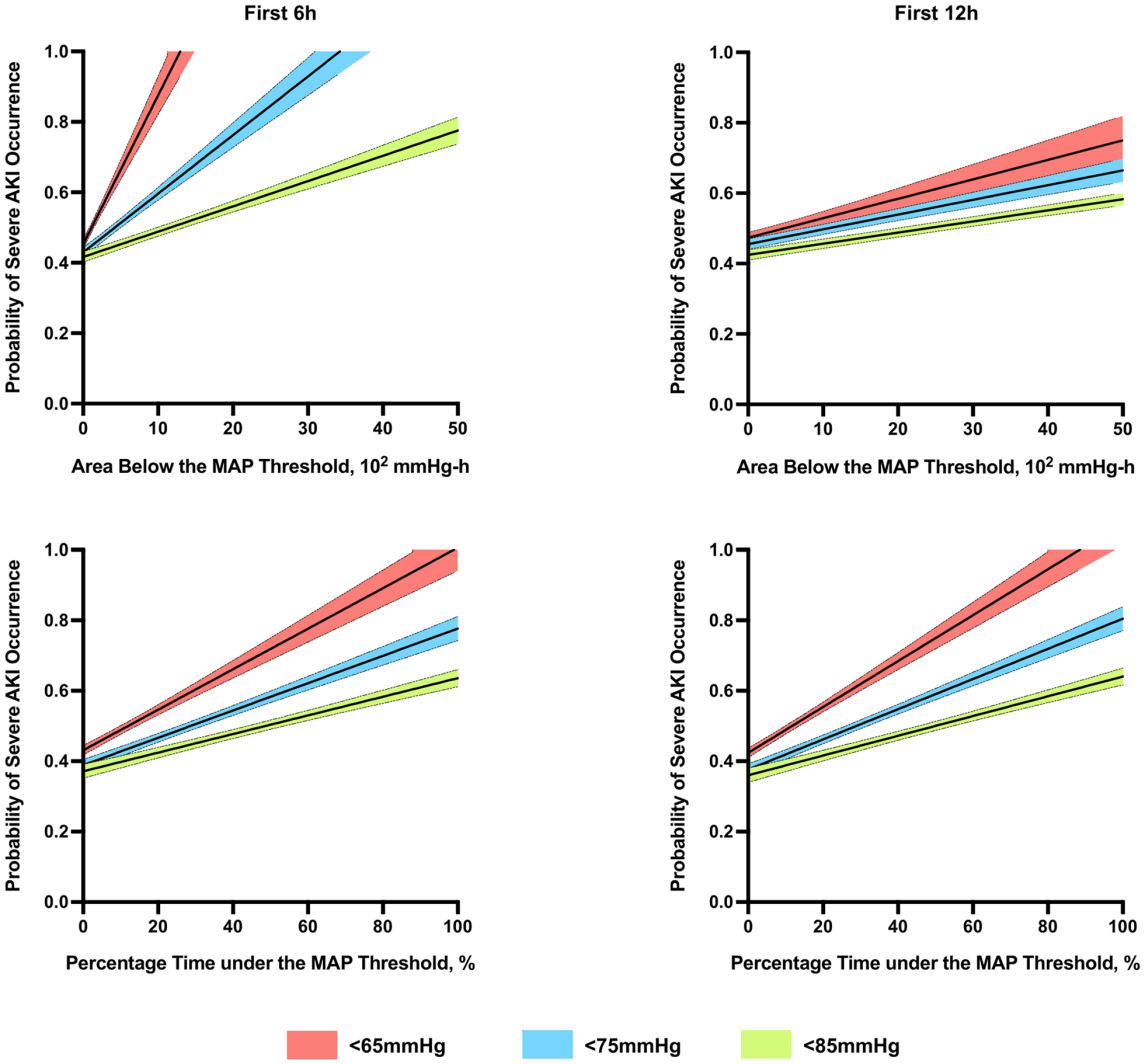
Predicted probabilities of severe acute kidney injury occurrence *versus* MAP-derived variables in patients with out-of-hospital cardiac arrest. Predicted multivariable-adjusted probabilities (with 95% confidence intervals) of severe acute kidney injury occurrence within the first 48 h after intensive care unit admission *versus* mean arterial pressure (MAP)-derived variables within the first 6 and 12 h after intensive care unit admission in patient with out-of-hospital cardiac arrest. All logistic regression models included one of the MAP-derived variables (percentage time spent under the MAP threshold or area below the MAP threshold in each model), age, gender, witness attendance, initial rhythm, epinephrine use during resuscitation, resuscitation durations, admission creatinine level, history of arterial hypertension and median norepinephrine dosage. *AKI* acute kidney injury

Next, we aimed to test the impact of the duration and severity of arterial hypotension under different MAP thresholds on severe AKI occurrence by analyzing the ABT. In univariate analysis, ABT were associated with severe AKI occurrence, regardless of the MAP threshold and the time period considered. After adjustment for covariates, ABT remained independently associated with severe AKI occurrence, regardless of the MAP threshold and the time considered (Table 2, Additional file 1: Tables S4–S9). The highest adjusted odds for developing severe AKI were observed while considering the first 6 h after ICU admission. Within the first 6 h, every 100 mmHg-h increase in ABT under MAP thresholds of 65, 75 and 85 mmHg increased severe AKI risk by 69% (OR = 1.69; 95% CI 1.26–2.26; *p* < 0.01), 13% (OR = 1.13; 95% CI 1.07–1.20; *p* < 0.01) and 4% (OR = 1.04; 95% CI 1.02–1.06; *p* < 0.01), respectively (Fig. 3).

### Percentage time under the MAP threshold and severe AKI occurrence

Then, in order to determine the most relevant MAP threshold in terms of severe AKI occurrence independently from the severity of arterial hypotension, we focused on the percentage time spent under the MAP thresholds. In univariate analysis, the percentage time spent under the MAP thresholds was associated with severe AKI occurrence, regardless of the MAP threshold and the time period considered. After adjustment for covariates, the percentage time spent under the MAP thresholds remained independently associated with severe AKI occurrence, regardless of the MAP and the time period considered (Table 2, Additional file 1: Tables S10–S14). The highest adjusted odds for developing severe AKI were observed while considering the first 6 h after ICU admission. Every 10% increase in percentage time spent under MAP thresholds of 65, 75 and 85 mmHg increased severe AKI risk by 19% (OR = 1.19; 95% CI 1.06–1.33; *p* < 0.01), 12% (OR = 1.12; 95% CI 1.04–1.19; *p* < 0.01) and 8% (OR = 1.08; 95% CI 1.02–1.14; *p* < 0.01), respectively (Fig. 3).

## Discussion

Severe AKI is frequent in patients with OHCA and represents a major burden because of its impact on treatment costs and on both short and long-term morbidity and mortality [6–8]. Using a large cohort of resuscitated patients with OHCA, we found that during the first 6 and 12 h after ICU admission, the percentage time spent under MAP thresholds ranging from 65 to 85 mmHg, as well as the corresponding ABT which reflects both the magnitude and duration of hypotension, were strongly associated with the occurrence of severe AKI, independently of the norepinephrine dosage and other demographic variables known to be associated with kidney failure [6]. The highest adjusted odds ratios for developing severe AKI were observed within the first 6 h after ICU admission.

To date, few studies have assessed the association between MAP level and renal function in patients with OHCA [6, 11]. In a post hoc analysis of the TTM-trial, Grand et al. reported that the MAP target (< 70 mmHg, 70–80 mmHg or > 80 mmHg) was inversely associated with estimated glomerular filtration rate and the need for RRT after OHCA [11]. However, these results might be interpreted with caution as MAP were recorded only at admission, then at 4, 12 and 20 h after admission, which limited the relevance of patients’ categorization given the non-negligible MAP variability during early post-OHCA period. Moreover, primary renal outcome was calculated with Cockcroft–Gault formula, which has been well-demonstrated to be unreliable in ICU patients [14].

To our knowledge, our study is one of the first investigating the effect of early MAP level on renal function in patients with OHCA by using data extracted from continuous MAP measurement, which provided a granular dataset allowing more precise analyses. We first demonstrated that the use of mean MAP value was lacking sensitivity and specificity to predict severe AKI occurrence, as attested by areas under ROC curves < 0.75 [15]. Moreover, analyzing the mean MAP value does not reflect the variations of MAP occurring within the early hours after ICU admission. We then tested the impact of both the duration and severity of arterial hypotension under different MAP thresholds, and demonstrated that the ABT were associated with severe AKI occurrence, regardless of the MAP threshold considered. Finally, since the association between the ABT corresponding to one predefined threshold and severe AKI occurrence may be influenced by the severity of arterial hypotension (i.e., the magnitude of deviation from the MAP threshold), we then focused on the percentage time in order to determine the most relevant MAP threshold in terms of severe AKI occurrence. Therefore, we demonstrated that the percentage time spent under the MAP threshold remained associated with the occurrence of severe AKI while considering a threshold as high as 85 mmHg, independently of the severity of arterial hypotension. Thus, for every 36 min within the first 6 h and every 72 min within the first 12 h after ICU admission spent under a MAP threshold of 85 mmHg, the risk of severe AKI occurrence increased by 8%.

Our results question current early hemodynamic strategy in patients with OHCA which aims to achieve a MAP level ≥ 65 mmHg [10], as recommended in patients with septic shock [16]. Indeed, in patients with septic shock, the SEPSIS-PAM study showed that increasing MAP target from 65–70 mmHg to 80–85 mmHg resulted in a decrease in the need for RRT in patients with chronic hypertension [17]. Our results suggest that targeting a MAP as high as 85 mmHg within the first 6 and 12 h after ICU admission could decrease the risk of severe AKI occurrence in the overall population of patients with OHCA. Interestingly, it has been well-demonstrated in animal studies that renal ischemia/reperfusion leads to a prolonged loss of renal autoregulation [18, 19]. Taken together, these results may suggest a potential benefit to target early high MAP level in patients with OHCA in order to prevent kidney damage.

Targeting higher MAP levels in patients with OHCA could also be beneficial besides the reduction of severe AKI occurrence [4, 20]. In a retrospective analysis of 122 OHCA patients, Russo et al. reported that higher ABT under a MAP threshold of 75 mmHg during the first 96 h was associated with increased rates of sever neurological dysfunction defined by a cerebral performance status ≥ 3 [21]. In a pre-planned analysis of a prospective cohort study, Roberts et al. showed that a mean MAP > 90 mmHg within the first 6 h was independently associated with a good neurological function at hospital discharge [22]. However, the COMACARE study did not find any difference in neurological outcome after OHCA when comparing two MAP levels (80–100 mmHg vs. 65–75 mmHg) [23]. Nevertheless, this study included a small number of patients and only used the neuron-specific enolase blood level at 48 h to define neurological outcome, without taking into account other markers [23]. Finally Ameloot et al. recently reported that targeting a MAP level between 80–85 and 100 mmHg was associated with smaller myocardial injury in post-cardiac arrest patients with shock after acute myocardial infraction [24]. Further investigations are warranted to assess the potential beneficial effect of early higher MAP on outcomes after OHCA [4].

While our study is the first to report an independent association between early MAP level and severe AKI occurrence in a large cohort of patients with OHCA by using continuous MAP measurement, we acknowledge some limitations. First, because these patients were well-characterized in terms of renal function and outcomes, we performed this post hoc analysis on a historical single-center cohort of our group [6]. However, our cohort’s demographics, management and mortality were similar to more recent cohorts of OHCA patients [25–27]. Second, the proportion of patients with severe AKI appeared to be higher in our study than in a previous report in OHCA survivors [28]*.* We assume that this large representation of severe AKI could be notably explained by more prolonged resuscitation durations and more severe admission parameters in our population. Third, we could not investigate the effects of all possible nephrotoxic agents commonly used in the ICU, including some antibiotics. Lastly, we did not have data regarding cardiac function and central venous pressure, which is an important parameter of the perfusion pressure. Such data would be interesting to consider in a prospective interventional trial.

## Conclusions

Both severity and duration of early arterial hypotension after ICU admission remained independently associated with severe AKI occurrence while considering a MAP threshold as high as 85 mmHg in patients with OHCA. Randomized controlled trials are direly needed to compare early higher MAP target *versus* standard care in this population.

## Supplementary Information

**Additional file 1: Table S1.** Comparison of included patients *versus *patients excluded for missing MAP values. **Table S2.** Multivariate analysis including mean MAP within the first 6h after ICU admission of factors associated with severe acute kidney injury occurrence within the first 48h after ICU admission. **Table S3.** Multivariate analysis including mean MAP within the first 12h after ICU admission of factors associated with severe acute kidney injury occurrence within the first 48h after ICU admission. **Table S4.** Multivariate analysis including ABT MAP < 65 mmHg within the first 6h after ICU admission of factors associated with severe acute kidney injury occurrence within the first 48h after ICU admission. **Table S5.** Multivariate analysis including ABT MAP < 75 mmHg within the first 6h after ICU admission of factors associated with severe acute kidney injury occurrence within the first 48h after ICU admission. **Table S6.** Multivariate analysis including ABT MAP < 85 mmHg within the first 6h after ICU admission of factors associated with severe acute kidney injury occurrence within the first 48h after ICU admission. **Table S7.** Multivariate analysis including ABT MAP < 65 mmHg within the first 12h after ICU admission of factors associated with severe acute kidney injury occurrence within the first 48h after ICU admission. **Table S8.** Multivariate analysis including ABT MAP < 75 mmHg within the first 12h after ICU admission of factors associated with severe acute kidney injury occurrence within the first 48h after ICU admission. **Table S9.** Multivariate analysis including ABT MAP < 85 mmHg within the first 12h after ICU admission of factors associated with severe acute kidney injury occurrence within the first 48h after ICU admission. **Table S10.** Multivariate analysis including the percentage time MAP < 65 mmHg within the first 6h after ICU admission of factors associated with severe acute kidney injury occurrence within the first 48h after ICU admission. **Table S11.** Multivariate analysis including the percentage time MAP < 75 mmHg within the first 6h after ICU admission of factors associated with severe acute kidney injury occurrence within the first 48h after ICU admission. **Table S12.** Multivariate analysis including the percentage time MAP < 85 mmHg within the first 6h after ICU admission of factors associated with severe acute kidney injury occurrence within the first 48h after ICU admission. **Table S13.** Multivariate analysis including the percentage time MAP < 65 mmHg within the first 12h after ICU admission of factors associated with severe acute kidney injury occurrence within the first 48h after ICU admission. **Table S14.** Multivariate analysis including the percentage time MAP < 75 mmHg within the first 12h after ICU admission of factors associated with severe acute kidney injury occurrence within the first 48h after ICU admission. **Table S15.** Multivariate analysis including the percentage time MAP < 85 mmHg within the first 12h after ICU admission of factors associated with severe acute kidney injury occurrence within the first 48h after ICU admission. **Figure S1.** Predictive ability of early mean MAP value for severe acute kidney injury occurrence within the first 48h after intensive care unit admission The predictive ability of the mean MAP value within the first 6 (A) and 12h (B) was studied by calculating ROC curves with their responding area under the curve (AUC).

## Data Availability

The datasets used in the current study are available from the corresponding author on reasonable request.

## Abbreviations

OHCA: Out-of-hospital cardiac arrest
AKI: Acute kidney injury
ICU: Intensive care unit
MAP: Mean arterial pressure
ROSC: Return of spontaneous circulation
KDIGO: Kidney Disease Improving Global Outcomes
RRT: Renal replacement therapy
ABT: Area below threshold
OR: Odds ratio
CI: Confidence interval
TTM: Targeted temperature management
