# Supplementary material for: Impact of early mean arterial pressure level on severe acute kidney injury occurrence after out-of-hospital cardiac arrest
Source: Ann Intensive Care. 2022 Jul 18;12:69. doi: 10.1186/s13613-022-01045-1 (PMC9288937; doi:10.1186/s13613-022-01045-1)
Supplement: Supplementary file 1 — Additional file 1: Table S1. Comparison of included patients versus patients excluded for missing MAP values. Table S2. Multivariate analysis including mean MAP within the first 6h after ICU admission of factors associated with severe acute kidney injury occurrence within the first 48h after ICU admission. Table S3. Multivariate analysis including mean MAP within the first 12h after ICU admission of factors associated with severe acute kidney injury occurrence within the first 48h after ICU admission. Table S4. Multivariate analysis including ABT MAP < 65 mmHg within the first 6h after ICU admission of factors associated with severe acute kidney injury occurrence within the first 48h after ICU admission. Table S5. Multivariate analysis including ABT MAP < 75 mmHg within the first 6h after ICU admission of factors associated with severe acute kidney injury occurrence within the first 48h after ICU admission. Table S6. Multivariate analysis including ABT MAP < 85 mmHg within the first 6h after ICU admission of factors associated with severe acute kidney injury occurrence within the first 48h after ICU admission. Table S7. Multivariate analysis including ABT MAP < 65 mmHg within the first 12h after ICU admission of factors associated with severe acute kidney injury occurrence within the first 48h after ICU admission. Table S8. Multivariate analysis including ABT MAP < 75 mmHg within the first 12h after ICU admission of factors associated with severe acute kidney injury occurrence within the first 48h after ICU admission. Table S9. Multivariate analysis including ABT MAP < 85 mmHg within the first 12h after ICU admission of factors associated with severe acute kidney injury occurrence within the first 48h after ICU admission. Table S10. Multivariate analysis including the percentage time MAP < 65 mmHg within the first 6h after ICU admission of factors associated with severe acute kidney injury occurrence within the first 48h after ICU admission. Table S11. Multivariate [file 13613_2022_1045_MOESM1_ESM.pdf]

Impact of early mean arterial pressure level on severe acute kidney injury occurrence after out-of-hospital cardiac arrest

Vincent DUPONT, MD; Anne-Sophie BONNET-LEBRUN, PhD; Alice BOILEVE, MD; Julien CHARPENTIER, MD; Jean-Paul MIRA, MD, PhD; Guillaume GERI, MD, PhD; Alain CARIOU, MD, PhD; Mathieu JOZWIAK, MD, PhD

Additional files

Additional Table 1. Comparison of included patients *versus* patients excluded for missing MAP values

Variables	Patients included (n=568)	Patients excluded for missing data (n=14)	*p* value
Male, n (%)	404 (71)	11 (79)	0.77
Age, years	59 (49-71)	63 (57-65)	0.19
BMI, kg/m^2	25 (23-28)	25 (22-27)	0.16
Hypertension, n (%)	219 (39)	8 (57)	0.17
Diabetes, n (%)	89 (16)	2 (14)	1.00
Witnessed CA, n (%)	491 (86)	14 (100)	0.23
Bystander CPR, n (%)	309 (54)	7 (50)	0.79
VF/VT, n (%)	324 (57)	7 (50)	0.60
Epinephrine use during CPR, mg	2.8±3.6	3.0±3.7	0.32
Time from collapse to CPR, min	4.9±5.5	4.0±4.4	0.25
Time from CPR to ROSC, min	17.8±11.9	16.0±8.6	0.21
Coronary angiogram, n (%)	408 (72)	13 (93)	0.13
Admission creatinine level, µmol/L	109±53	115±36	0.27
Admission lactate level, mmol/L	5.0±3.6	6.0±5.3	0.17
Diuresis within the first 24 h, mL/kg/h	0.9±0.8	0.9±1.0	0.27
Severe AKI within 48h after admission	274 (48)	6 (43)	0.79
Day-30 mortality, n (%)	328 (58)	7 (50)	0.59

Data are expressed as mean±standard deviation, median [interquartile] or number (%). BMI: body mass index, CA: cardiac arrest, CPR: cardiopulmonary resuscitation, VF: ventricular fibrillation, VT: ventricular tachycardia, ROSC: return of spontaneous circulation, AKI: acute kidney injury.

Additional Table 2. Multivariate analysis including mean MAP within the first 6h after ICU admission of factors associated with severe acute kidney injury occurrence within the first 48h after ICU admission

	β coefficient	Standard error	Adjusted OR [95%CI]	*p* value
Admission creatinine level, μmol/L	0.02	<0.01	1.02 [1.01-1.02]	<0.01
Epinephrine use during CPR, mg	0.02	0.04	1.02 [0.96-1.10]	0.50
Time from collapse to CPR, min	0.03	0.02	1.03 [0.98-1.08]	0.21
Time from CPR to ROSC, min	0.03	0.01	1.03 [1.00-1.05]	0.03
Age, years	0.02	0.01	1.02 [1.00-1.04]	0.03
Median norepinephrine dosage within first 6h, μg/kg/min	0.52	0.62	1.68 [0.50-5.62]	0.40
Mean MAP value within first 6h	<0.01	0.01	1.00 [0.98-1.02]	0.93
Male	0.75	0.28	2.12 [1.24-3.62]	<0.01
Hypertension	0.51	0.26	1.66 [1.00-2.77]	0.05
Witness attendance	1.12	0.50	3.07 [1.15-8.21]	0.03
Shockable rhythm	0.29	0.25	1.34 [0.81-2.20]	0.26

MAP: mean arterial pressure, ICU: intensive care unit, OR: odds ratio, CI: confidence interval, CPR: cardiopulmonary resuscitation.

Additional Table 3. Multivariate analysis including mean MAP within the first 12h after ICU admission of factors associated with severe acute kidney injury occurrence within the first 48h after ICU admission

	β coefficient	Standard error	Adjusted OR [95%CI]	*p* value
Admission creatinine level, µmol/L	0.02	<0.01	1.02 [1.01-1.02]	<0.01
Epinephrine use during CPR, mg	0.03	0.04	1.03 [0.96-1.10]	0.50
Time from collapse to CPR, min	0.03	0.02	1.03 [0.98-1.08]	0.21
Time from CPR to ROSC, min	0.03	0.01	1.03 [1.00-1.05]	0.03
Age, years	0.02	0.01	1.02 [1.00-1.04]	0.04
Median norepinephrine dosage within first 12h, µg/kg/min	0.40	0.55	1.49 [0.50-4.38]	0.47
Mean MAP value within first 12h	0.01	0.01	1.01 [0.99-1.03]	0.50
Male	0.76	0.27	2.13 [1.25-3.64]	<0.01
Hypertension	0.51	0.26	1.67 [1.00-2.78]	0.05
Witness attendance	1.14	0.50	3.12 [1.17-8.32]	0.02
Shockable rhythm	0.29	0.26	1.34 [0.81-2.21]	0.25

MAP: mean arterial pressure, ICU: intensive care unit, OR: odds ratio, CI: confidence interval, CPR: cardiopulmonary resuscitation.

Additional Table 4. Multivariate analysis including ABT MAP < 65 mmHg within the first 6h after ICU admission of factors associated with severe acute kidney injury occurrence within the first 48h after ICU admission

	β coefficient	Standard error	Adjusted OR [95%CI]	*p* value
Admission creatinine level, µmol/L	0.01	<0.01	1.02 [1.01-1.02]	<0.01
Epinephrine use during CPR, mg	<0.01	0.04	1.00 [0.93-1.08]	0.94
Time from collapse to CPR, min	0.05	0.02	1.06 [1.01-1.10]	0.02
Time from CPR to ROSC, min	0.03	0.01	1.03 [1.01-1.05]	0.01
Age, years	0.02	0.01	1.02 [1.00-1.03]	0.06
Median norepinephrine dosage within first 6h, µg/kg/min	0.62	0.53	1.86 [0.66-5.22]	0.24
ABT MAP <65mmHg within first 6h, 10^2mmHg-h	0.52	0.15	1.69 [1.26-2.26]	<0.01
Male	0.81	0.26	2.26 [1.35-3.77]	<0.01
Hypertension	0.46	0.24	1.58 [0.99-2.53]	0.06
Witness attendance	0.87	0.46	2.39 [0.96-5.93]	0.06
Shockable rhythm	0.27	0.24	1.31 [0.83-2.09]	0.25

MAP: mean arterial pressure, ICU: intensive care unit, OR: odds ratio, CI: confidence interval, CPR: cardiopulmonary resuscitation, ABT: area below threshold.

Additional Table 5. Multivariate analysis including ABT MAP < 75 mmHg within the first 6h after ICU admission of factors associated with severe acute kidney injury occurrence within the first 48h after ICU admission

	β coefficient	Standard error	Adjusted OR [95%CI]	*p* value
Admission creatinine level, μmol/L	0.02	<0.01	1.02 [1.01-1.02]	<0.01
Epinephrine use during CPR, mg	0.01	0.04	1.01 [0.94-1.08]	0.76
Time from collapse to CPR, min	0.06	0.02	1.06 [1.01-1.11]	0.01
Time from CPR to ROSC, min	0.03	0.01	1.03 [1.00-1.05]	0.02
Age, years	0.02	0.01	1.01 [1.00-1.03]	0.06
Median norepinephrine dosage within first 6h, μg/kg/min	0.61	0.53	1.85 [0.65-5.26]	0.25
ABT MAP <75mmHg within first 6h, 10^2mmHg-h	0.12	0.03	1.13 [1.07-1.20]	<0.01
Male	0.82	0.26	2.26 [1.35-3.77]	<0.01
Hypertension	0.44	0.24	1.56 [0.97-2.49]	0.06
Witness attendance	0.87	0.46	2.39 [0.97-5.88]	0.06
Shockable rhythm	0.26	0.24	1.30 [0.82-2.07]	0.27

MAP: mean arterial pressure, ICU: intensive care unit, OR: odds ratio, CI: confidence interval, CPR: cardiopulmonary resuscitation, ABT: area below threshold.

Additional Table 6. Multivariate analysis including ABT MAP < 85 mmHg within the first 6h after ICU admission of factors associated with severe acute kidney injury occurrence within the first 48h after ICU admission

	β coefficient	Standard error	Adjusted OR [95%CI]	*p* value
Admission creatinine level, μmol/L	0.01	<0.01	1.01 [1.01-1.02]	<0.01
Epinephrine use during CPR, mg	0.02	0.03	1.02 [0.95-1.09]	0.61
Time from collapse to CPR, min	0.06	0.02	1.06 [1.01-1.11]	0.01
Time from CPR to ROSC, min	0.03	0.01	1.03 [1.01-1.05]	0.01
Age, years	0.02	0.01	1.02 [1.00-1.03]	0.05
Median norepinephrine dosage within first 6h, μg/kg/min	0.70	0.54	2.01 [0.70-5.76]	0.19
ABT MAP <85mmHg within first 6h, 10^2mmHg-h	0.04	0.01	1.04 [1.02-1.06]	<0.01
Male	0.85	0.26	2.25 [1.35-3.74]	<0.01
Hypertension	0.44	0.24	1.55 [0.97-2.48]	0.07
Witness attendance	0.88	0.46	2.41 [0.98-5.91]	0.06
Shockable rhythm	0.27	0.24	1.30 [0.82-2.07]	0.26

MAP: mean arterial pressure, ICU: intensive care unit, OR: odds ratio, CI: confidence interval, CPR: cardiopulmonary resuscitation, ABT: area below threshold.

Additional Table 7. Multivariate analysis including ABT MAP < 65 mmHg within the first 12h after ICU admission of factors associated with severe acute kidney injury occurrence within the first 48h after ICU admission

	β coefficient	Standard error	Adjusted OR [95%CI]	*p* value
Admission creatinine level, μmol/L	0.01	<0.01	1.02 [1.01-1.02]	<0.01
Epinephrine use during CPR, mg	<0.01	0.04	1.00 [0.93-1.08]	0.95
Time from collapse to CPR, min	0.06	0.02	1.06 [1.01-1.11]	0.01
Time from CPR to ROSC, min	0.03	0.01	1.03 [1.01-1.05]	0.01
Age, years	0.01	0.01	1.01 [1.00-1.03]	0.09
Median norepinephrine dosage within first 12h, μg/kg/min	0.55	0.51	1.74 [0.63-4.76]	0.28
ABT MAP <65mmHg within first 12h, 10^2mmHg-h	0.39	0.10	1.48 [1.21-1.81]	<0.01
Male	0.82	0.26	2.28 [1.36-3.82]	<0.01
Hypertension	0.46	0.24	1.59 [0.99-2.55]	0.05
Witness attendance	0.90	0.46	2.46 [0.99-6.11]	0.05
Shockable rhythm	0.30	0.24	1.34 [0.84-2.14]	0.22

MAP: mean arterial pressure, ICU: intensive care unit, OR: odds ratio, CI: confidence interval, CPR: cardiopulmonary resuscitation, ABT: area below threshold.

Additional Table 8. Multivariate analysis including ABT MAP < 75 mmHg within the first 12h after ICU admission of factors associated with severe acute kidney injury occurrence within the first 48h after ICU admission

	β coefficient	Standard error	Adjusted OR [95%CI]	*p* value
Admission creatinine level, μmol/L	0.02	<0.01	1.02 [1.01-1.02]	<0.01
Epinephrine use during CPR, mg	<0.01	0.04	1.00 [0.93-1.08]	0.99
Time from collapse to CPR, min	0.06	0.02	1.06 [1.02-1.11]	0.01
Time from CPR to ROSC, min	0.03	0.01	1.03 [1.01-1.05]	0.01
Age, years	0.01	0.01	1.01 [1.00-1.03]	0.10
Median norepinephrine dosage within first 12h, μg/kg/min	0.60	0.52	1.83 [0.67-5.02]	0.24
ABT MAP <75mmHg within first 12h, 10^2mmHg-h	0.07	0.02	1.07 [1.04-1.10]	<0.01
Male	0.80	0.26	2.21 [1.32-3.71]	<0.01
Hypertension	0.48	0.24	1.62 [1.01-2.60]	0.05
Witness attendance	0.96	0.46	2.61 [1.06-6.39]	0.04
Shockable rhythm	0.31	0.24	1.37 [0.86-2.18]	0.19

MAP: mean arterial pressure, ICU: intensive care unit, OR: odds ratio, CI: confidence interval, CPR: cardiopulmonary resuscitation, ABT: area below threshold.

Additional Table 9. Multivariate analysis including ABT MAP < 85 mmHg within the first 12h after ICU admission of factors associated with severe acute kidney injury occurrence within the first 48h after ICU admission

	β coefficient	Standard error	Adjusted OR [95%CI]	*p* value
Admission creatinine level, μmol/L	0.02	<0.01	1.02 [1.01-1.02]	<0.01
Epinephrine use during CPR, mg	<0.01	0.04	1.00 [0.93-1.08]	0.95
Time from collapse to CPR, min	0.06	0.01	1.06 [1.01-1.11]	0.01
Time from CPR to ROSC, min	0.03	0.01	1.03 [1.01-1.05]	0.01
Age, years	0.01	0.01	1.01 [1.00-1.03]	0.08
Median norepinephrine dosage within first 12h, μg/kg/min	0.56	0.52	1.74 [0.63-4.80]	0.28
ABT MAP <85mmHg within first 12h, 10^2mmHg-h	0.02	<0.01	1.02 [1.01-1.03]	<0.01
Male	0.82	0.26	2.28 [1.37-3.80]	<0.01
Hypertension	0.48	0.24	1.62 [1.01-2.59]	0.05
Witness attendance	0.97	0.46	2.64 [1.08-6.47]	0.03
Shockable rhythm	0.29	0.24	1.34 [0.84-2.13]	0.22

MAP: mean arterial pressure, ICU: intensive care unit, OR: odds ratio, CI: confidence interval, CPR: cardiopulmonary resuscitation, ABT: area below threshold.

Additional Table 10. Multivariate analysis including the percentage time MAP < 65 mmHg within the first 6h after ICU admission of factors associated with severe acute kidney injury occurrence within the first 48h after ICU admission

	β coefficient	Standard error	Adjusted OR [95%CI]	*p* value
Admission creatinine level, μmol/L	0.02	<0.01	1.02 [1.01-1.02]	<0.01
Epinephrine use during CPR, mg	<0.01	0.04	1.00 [0.94-1.08]	0.91
Time from collapse to CPR, min	0.05	0.02	1.06 [1.01-1.10]	0.02
Time from CPR to ROSC, min	0.03	0.01	1.03 [1.01-1.05]	0.01
Age, years	0.01	0.01	1.01 [1.00-1.03]	0.08
Median norepinephrine dosage within first 6h, μg/kg/min	0.74	0.54	2.09 [0.73-5.94]	0.17
Percentage time MAP <65mmHg within first 6h, 10%	0.19	0.07	1.19 [1.06-1.33]	<0.01
Male	0.85	0.26	2.33 [1.41-3.85]	<0.01
Hypertension	0.47	0.24	1.60 [1.00-2.56]	0.05
Witness attendance	0.92	0.46	2.52 [1.03-6.17]	0.04
Shockable rhythm	0.24	0.23	1.27 [0.80-2.00]	0.31

MAP: mean arterial pressure, ICU: intensive care unit, OR: odds ratio, CI: confidence interval, CPR: cardiopulmonary resuscitation.

Additional Table 11. Multivariate analysis including the percentage time MAP < 75 mmHg within the first 6h after ICU admission of factors associated with severe acute kidney injury occurrence within the first 48h after ICU admission

	β coefficient	Standard error	Adjusted OR [95%CI]	*p* value
Admission creatinine level, μmol/L	0.01	<0.01	1.01 [1.01-1.02]	<0.01
Epinephrine use during CPR, mg	<0.01	0.03	1.00 [0.94-1.08]	0.86
Time from collapse to CPR, min	0.06	0.02	1.06 [1.01-1.11]	0.01
Time from CPR to ROSC, min	0.03	0.01	1.03 [1.01-1.05]	0.01
Age, years	0.01	0.01	1.02 [1.00-1.03]	0.06
Median norepinephrine dosage within first 6h, μg/kg/min	0.64	0.55	1.89 [0.64-5.61]	0.25
Percentage time MAP <75mmHg within first 6h, 10%	0.12	0.04	1.12 [1.04-1.19]	<0.01
Male	0.83	0.26	2.29 [1.39-3.78]	<0.01
Hypertension	0.43	0.24	1.54 [0.96-2.46]	0.07
Witness attendance	0.90	0.46	2.45 [1.00-6.00]	0.05
Shockable rhythm	0.23	0.23	1.26 [0.80-1.99]	0.32

MAP: mean arterial pressure, ICU: intensive care unit, OR: odds ratio, CI: confidence interval, CPR: cardiopulmonary resuscitation.

Additional Table 12. Multivariate analysis including the percentage time MAP < 85 mmHg within the first 6h after ICU admission of factors associated with severe acute kidney injury occurrence within the first 48h after ICU admission

	β coefficient	Standard error	Adjusted OR [95%CI]	*p* value
Admission creatinine level, μmol/L	0.01	<0.01	1.02 [1.01-1.02]	<0.01
Epinephrine use during CPR, mg	0.01	0.03	1.01 [0.95-1.08]	0.70
Time from collapse to CPR, min	0.05	0.02	1.06 [1.01-1.11]	0.02
Time from CPR to ROSC, min	0.03	0.01	1.03 [1.01-1.05]	0.01
Age, years	0.02	0.01	1.02 [1.00-1.03]	0.05
Median norepinephrine dosage within first 6h, μg/kg/min	0.72	0.55	2.06 [0.70-6.07]	0.19
Percentage time MAP <85mmHg within first 6h, 10%	0.08	0.03	1.08 [1.02-1.14]	<0.01
Male	0.84	0.26	2.32 [1.41-3.82]	<0.01
Hypertension	0.45	0.24	1.56 [0.98-2.49]	0.06
Witness attendance	0.88	0.46	2.41 [0.99-5.87]	0.05
Shockable rhythm	0.25	0.23	1.28 [0.82-2.02]	0.28

MAP: mean arterial pressure, ICU: intensive care unit, OR: odds ratio, CI: confidence interval, CPR: cardiopulmonary resuscitation.

Additional Table 13. Multivariate analysis including the percentage time MAP < 65 mmHg within the first 12h after ICU admission of factors associated with severe acute kidney injury occurrence within the first 48h after ICU admission

	β coefficient	Standard error	Adjusted OR [95%CI]	*p* value
Admission creatinine level, µmol/L	0.02	<0.01	1.02 [1.01-1.02]	<0.01
Epinephrine use during CPR, mg	0.01	0.04	1.01 [0.94-1.08]	0.87
Time from collapse to CPR, min	0.06	0.02	1.06 [1.01-1.11]	0.01
Time from CPR to ROSC, min	0.03	0.01	1.03 [1.01-1.05]	0.01
Age, years	0.01	0.01	1.01 [1.00-1.03]	0.10
Median norepinephrine dosage within first 12h, µg/kg/min	0.61	0.50	1.84 [0.69-4.88]	0.22
Percentage time MAP <65mmHg within first 12h, 10%	0.23	0.07	1.23 [1.09-1.37]	<0.01
Male	0.86	0.26	2.35 [1.42-3.89]	<0.01
Hypertension	0.49	0.24	1.63 [1.02-2.61]	0.04
Witness attendance	0.96	0.46	2.61 [1.06-6.40]	0.04
Shockable rhythm	0.21	0.23	1.24 [0.78-1.96]	0.36

MAP: mean arterial pressure, ICU: intensive care unit, OR: odds ratio, CI: confidence interval, CPR: cardiopulmonary resuscitation.

Additional Table 14. Multivariate analysis including the percentage time MAP < 75 mmHg within the first 12h after ICU admission of factors associated with severe acute kidney injury occurrence within the first 48h after ICU admission

	β coefficient	Standard error	Adjusted OR [95%CI]	*p* value
Admission creatinine level, μmol/L	0.02	<0.01	1.01 [1.01-1.02]	<0.01
Epinephrine use during CPR, mg	0.01	0.03	1.01 [0.94-1.08]	0.85
Time from collapse to CPR, min	0.06	0.02	1.06 [1.01-1.11]	0.01
Time from CPR to ROSC, min	0.03	0.01	1.03 [1.01-1.05]	0.01
Age, years	0.01	0.01	1.01 [1.00-1.03]	0.08
Median norepinephrine dosage within first 12h, μg/kg/min	0.56	0.51	1.75 [0.64-4.78]	0.27
Percentage time MAP <75mmHg within first 12h, 10%	0.14	0.04	1.14 [1.06-1.22]	<0.01
Male	0.83	0.26	2.29 [1.38-3.79]	<0.01
Hypertension	0.43	0.24	1.54 [0.96-2.47]	0.07
Witness attendance	0.94	0.46	2.57 [1.05-6.30]	0.04
Shockable rhythm	0.23	0.23	1.25 [0.79-1.98]	0.33

MAP: mean arterial pressure, ICU: intensive care unit, OR: odds ratio, CI: confidence interval, CPR: cardiopulmonary resuscitation.

Additional Table 14. Multivariate analysis including the percentage time MAP < 85 mmHg within the first 12h after ICU admission of factors associated with severe acute kidney injury occurrence within the first 48h after ICU admission

	β coefficient	Standard error	Adjusted OR [95%CI]	*p* value
Admission creatinine level, µmol/L	0.02	<0.01	1.01 [1.01-1.02]	<0.01
Epinephrine use during CPR, mg	0.01	0.03	1.01 [0.95-1.08]	0.70
Time from collapse to CPR, min	0.06	0.02	1.06 [1.01-1.11]	0.01
Time from CPR to ROSC, min	0.03	0.01	1.03 [1.01-1.05]	0.01
Age, years	0.01	0.01	1.01 [1.00-1.03]	0.06
Median norepinephrine dosage within first 12h, µg/kg/min	0.58	0.51	1.78 [0.66-4.79]	0.25
Percentage time MAP <85mmHg within first 12h, 10%	0.08	0.03	1.08 [1.02-1.14]	<0.01
Male	0.86	0.25	2.37 [1.44-3.90]	<0.01
Hypertension	0.46	0.24	1.58 [0.99-2.52]	0.06
Witness attendance	0.92	0.46	2.50 [1.02-6.08]	0.04
Shockable rhythm	0.25	0.23	1.28 [0.82-2.02]	0.28

MAP: mean arterial pressure, ICU: intensive care unit, OR: odds ratio, CI: confidence interval, CPR: cardiopulmonary resuscitation.

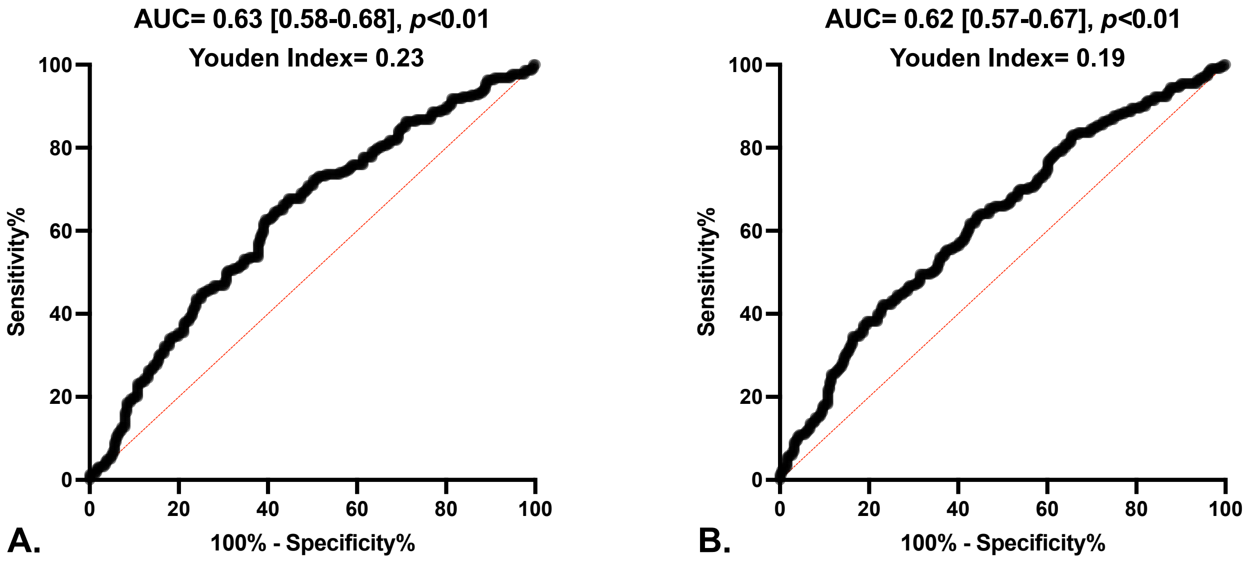

Additional Figure 1. Predictive ability of early mean MAP value for severe acute kidney injury occurrence within the first 48h after intensive care unit admission

The predictive ability of the mean MAP value within the first 6 (A) and 12h (B) was studied by calculating ROC curves with their responding area under the curve (AUC).
